# A Graph-Centric Approach for Metagenome-Guided Peptide and Protein Identification in Metaproteomics

**DOI:** 10.1371/journal.pcbi.1005224

**Published:** 2016-12-05

**Authors:** Haixu Tang, Sujun Li, Yuzhen Ye

**Affiliations:** School of Informatics and Computing, Indiana University, Bloomington, Indiana, United States of America; Thermo Fisher Scientific, GERMANY

## Abstract

Metaproteomic studies adopt the common bottom-up proteomics approach to investigate the protein composition and the dynamics of protein expression in microbial communities. When matched metagenomic and/or metatranscriptomic data of the microbial communities are available, metaproteomic data analyses often employ a metagenome-guided approach, in which complete or fragmental protein-coding genes are first directly predicted from metagenomic (and/or metatranscriptomic) sequences or from their assemblies, and the resulting protein sequences are then used as the reference database for peptide/protein identification from MS/MS spectra. This approach is often limited because protein coding genes predicted from metagenomes are incomplete and fragmental. In this paper, we present a graph-centric approach to improving metagenome-guided peptide and protein identification in metaproteomics. Our method exploits the *de Bruijn* graph structure reported by metagenome assembly algorithms to generate a comprehensive database of protein sequences encoded in the community. We tested our method using several public metaproteomic datasets with matched metagenomic and metatranscriptomic sequencing data acquired from complex microbial communities in a biological wastewater treatment plant. The results showed that many more peptides and proteins can be identified when assembly graphs were utilized, improving the characterization of the proteins expressed in the microbial communities. The additional proteins we identified contribute to the characterization of important pathways such as those involved in degradation of chemical hazards. Our tools are released as open-source software on github at https://github.com/COL-IU/Graph2Pro.

## Author Summary

In recent years, meta-omic (including metatranscriptomic and metaproteomic) techniques have been adopted as complementary approaches to metagenomic sequencing to study functional characteristics and dynamics of microbial communities, aiming at a holistic understanding of a community to respond to the changes in the environment. Currently, metaproteomic data are largely analyzed using the bioinformatics tools originally designed in bottom-up proteomics. In particular, recent metaproteomic studies employed a metagenome-guided approach, in which complete or fragmental protein-coding genes were first predicted from metagenomic sequences (i.e., contigs or scaffolds), acquired from the matched community samples, and predicted protein sequences were then used in peptide identification. A key challenge of this approach is that the protein coding genes predicted from assembled metagenomic contigs can be incomplete and fragmented due to the complexity of metagenomic samples and the short reads length in metagenomic sequencing. To address this issue, in this paper, we present a graph-centric approach that exploits the *de bruijn* graph structure reported by metagenome assembly algorithms to improve metagenome-guided peptide and protein identification in metaproteomics. We show that our method can identify much more peptides and proteins, improving the characterization of the proteins expressed in the microbial communities.

## Introduction

Microbiome studies have produced massive metagenomic data, and more recently other meta-omics including metatranscriptomic and metaproteomic data [1]. Analyses of these data reveal insights into the composition, function and regulatory characteristics of the microbial communities associated with different ecosystems, habitats and hosts [2–8]. While metagenomic sequencing reveal important properties of microbial communities, other meta-omic (e.g., metatranscriptomic [9, 10] and metaproteomic [11–13]) techniques can provide additional insights, in particular on functional characteristics, such as gene activities, their regulation mechanisms and the dynamics of microbial communities, to understand how microbial organisms work as a community to respond to the changes in their environment, e.g., the health condition of the host of human microbiome [14–16]. Current metatranscriptomic and metaproteomic studies often directly adopt protocols originally developed for the transcriptomic and proteomic studies of model bacterial species; for examples, many metatranscriptomic projects exploited the bacterial RNA-seq protocol [17, 18], while most metaproteomic studies applied the common bottom-up proteomics approach, in which the proteins extracted from community samples are first tryptically digested and then analyzed by using one-dimensional or two-dimensional liquid chromatography tandem mass spectrometry (LC-MS/MS) [19–24].

Similarly, metaproteomic data are analyzed using the bioinformatics approaches used in bottom-up proteomics. Specifically, the first step of metaproteomic data analysis is the peptide identification, achieved by searching MS/MS spectra from an LC-MS/MS experiment against the tryptic peptides *in silico* digested from a target database of proteins that are potentially present in the metaproteomic sample. Many peptide search engines have been developed for this purpose in the proteomics field, including commonly used tools such as Mascot [25], Sequest [26], X!Tandem [27], InSPEct [28] and MSGF+ [29]. Their applications in metaproteomics rely on the pre-assembly of a protein database. Early metaproteomic studies used the collection of proteins encoded by fully sequenced bacterial genomes that likely live in the environment (e.g., human gut [11]) as the target database. This collection may be largely incomplete, e.g., a large fraction (10%-34%) of genes from HMP [30] or MetaHIT [31] shotgun sequencing are completely novel [6]. As a result, more recent metaproteomic studies employed a metagenome-guided approach, in which complete or fragmental protein-coding genes were first predicted from metagenomic sequences (i.e., contigs or scaffolds), acquired from the matched community samples, and predicted protein sequences were then used in peptide identification [24]. Several software tools have been developed for protein coding gene prediction from metagenomic sequences, including MetaGeneMark [32] and our own software FragGeneScan [33, 34]. A key challenge of this approach is that the protein coding genes predicted from assembled metagenomic contigs can be incomplete and fragmented due to the complexity of metagenomic samples and the short reads length in metagenomic sequencing. As the linear representations of contigs and scaffolds in metagenome assembly do not capture their putative connections, the short contigs contain only gene fragments, and even long contigs contain broken genes at their ends. As a result, the target peptides collected in this manner may miss many full-length tryptic peptides that are potentially observed in the metaproteomic experiments.

To alleviate the peptide/protein identification problem caused by incomplete/fragmental reference proteins, we propose a graph-centric approach to improving metagenome-guided peptide identification in metaproteomics. Many short read assemblers, including those commonly used for metagenome assembly such as Velvet [35], SOAPdenovo [36], MegaHIT [37] and SPAdes [38], employed the *de Bruijn* graph [39, 40] as the core data structure, in which each edge represents an assembled unique sequence from metagenomic reads (i.e., the contigs), and the graph structure represents the ambiguous connections between contigs that cannot be resolved by using sequencing reads. Some assemblers including SOAPdenovo [41] and metaSPAdes [42] report the *de Bruijn* graph of the assembly along with contigs. As demonstrated in our previous work, by exploiting the *de Bruijn* graph structure in metagenome assembly, we can reconstruct longer and more complete transcript sequences from short metatranscriptomic reads than the straightforward approach based solely on contigs [43]. Here, we attempt to predict protein coding genes directly from the sequences in the *de Bruijn* graph, including the proteins that span multiple edges in the graph, to expand the target protein database for metaproteomic data analysis. We implemented an algorithm that takes as input the *de Bruijn* graph of a metagenome assembly, traverses the graph in a depth-first search (DFS) fashion, and outputs a target database consisting of the tryptic peptides in all putative open reading frames (ORFs) encountered during the traversal. In the following step, the identified tryptic peptides were used to retrieve potential protein sequences by traversing the graph for the second time. Using three metaproteomic datasets with matched metagenomic sequencing data, we show that much more peptides and proteins can be identified when the targeted database is constructed from graph structures of matched metagenomic sequences than those from the database only consisting of proteins predicted from contigs, indicating the metagenome-guided graph-centric approach can improve the peptide and proteins identification in metaproteomics.

## Materials and Methods

### Overview

**Fig 1:**
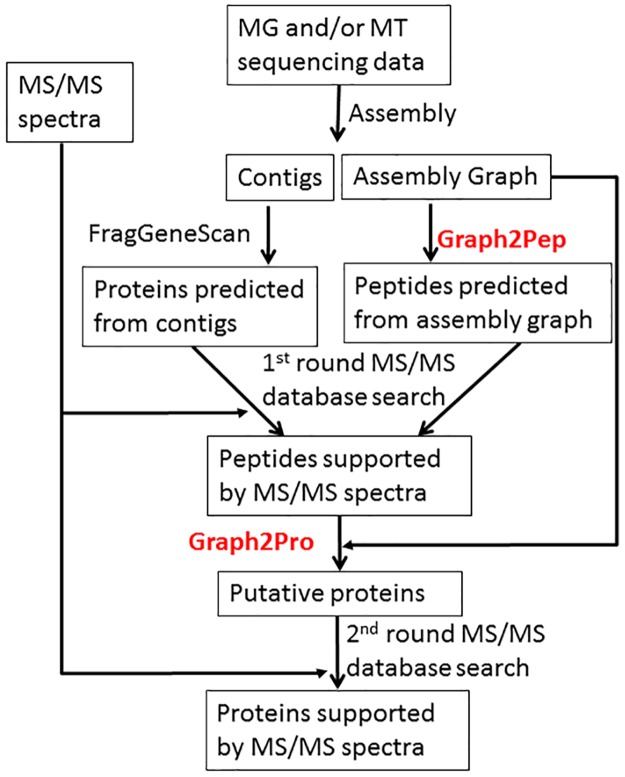
An overview for protein identification using metaproteomic data, with metagenomic (MG) sequencing and metatranscriptomic (MT) data obtained from matched samples. We report two novel graph traversal algorithms (Graph2Pep and Graph2Pro, highlighted in red in the figure) to extract peptides and proteins from the *de Bruijn* graph representation of metagenome/metatranscriptome assemblies, respectively. We note the same pipeline can be applied when only matched metagenomic or metatranscriptomic data (but not both) is available, in which the graph algorithms will be applied to the assembly graph of metagenome (or metatranscriptome).

As illustrated in Fig 1, we developed a pipeline for protein identification from metaproteomic data when metagenomic and metatranscriptomic data are acquired from matched samples. The pipeline exploits the maximum information available when both metagenomic and metatranscriptomic data are obtained from matched samples, and attempts to address the objective of protein identification in metaproteomics.

The pipeline is particularly useful when the depth of metagenomic and metatranscriptomic sequencing are not sufficiently high, and thus they complement to each other to provide a comprehensive coverage of the whole set of genes encoded in the metagenome. In this pipeline, we first assemble the metagenomic and metatranscriptomic sequencing data together (note that because there is no split gene structures in bacterial genes, metatranscriptomic sequencing reads represent contiguous segments in corresponding bacterial genomes in the same manner as metagenomic reads), and the resulting assembly (denoted as *Assembly-Combined*) are used to construct the target protein database for protein identification.

We emphasize that in the pipeline, the metagenome/metatranscriptome assembly is represented as *de Bruijn* graphs instead of a collection of contig sequences as used in conventional methods. As a result, peptide/protein sequences are extracted from the *de Bruijn* graphs, and thus may span multiple edges (contigs) in the graph. In order to retain the *de Bruijn* graph representation in the assembly, we take the SOAPdenovo assembly algorithm [41] as an example in this paper, which reports the *de Bruijn* graph structures in addition to the contig sequences in the assembly. Other assemblers can also be used in our pipeline, as long as they report graph structures of the assembly. Below we will present software tools (Graph2Pep and Graph2Pro) to extract peptides/proteins from the *de Bruijn* graphs of metagenome and/or metatranscriptome assembly.

### Prediction of peptides and protein sequences from *de Bruijn* graphs

To utilize *de Bruijn* graphs of metagenome assembly for protein identification, we use a two-step strategy: in the first step, all putative tryptic peptides are predicted from the *de Bruijn* graph, while in the second step, full-length protein sequences are predicted to cover the whole set of tryptic peptides identified from the initial database searching results of the metaproteomic data. This way, we will not overburden the MS/MS spectra identification with excessive and potentially error-prone reference protein sequences that could be predicted from the graphs.

**Fig 2:**
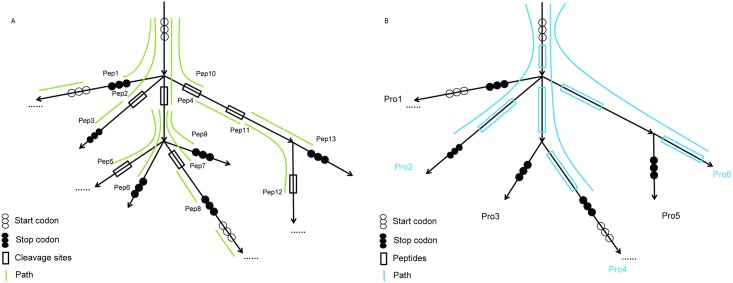
A schematic illustration of the graph traversal algorithms for extracting tryptic peptides (Graph2Pep; A) and proteins (Graph2Pro; B) from the *de Bruijn* graph assembly.

As illustrated in Fig 2, our core algorithms (Graph2Pep and Graph2Pro) for peptide/protein identification from graphs both take as input a *contracted* *de Bruijn* graph, a directed graph reported by a fragment assembly algorithm (such as SOAPdenovo), in which each vertex represents a k-mer, and each edge represents a DNA sequence resulting from the collapse of the one-in-one-out k-mers between the two terminal vertices. Because both DNA strands are represented in the graph, it has a symmetric property: each edge (and vertex) has a counterpart that represent the reverse complement of the DNA sequence represented by the edge (and vertex); when an edge represents a palindromic sequence, its counterpart is itself.

The combined set of tryptic peptides, including those predicted from long edges and those extracted from one or more short edges in the graph (by Graph2Pep), are used as the target database for peptide identification in the metaproteomic data by using a peptide search engine (such as MSGF+ as used here). Note that this step is not going to generate the final report of protein identification; instead, it will produce a collection of tryptic peptides that are encoded in the *de Bruijn* graph assembly, and are likely to be present in the sample. Therefore, we can use a less stringent criterion to filter peptide identifications (i.e., by using a relatively low FDR threshold 5% in this work) so that more putative peptides can be taken into consideration when we attempt to construct the target database of potential proteins in the sample (using Graph2Pro; see below) for the second (and final) step of protein identification.

#### The Graph2Pep algorithm

The first algorithm (Graph2Pep) attempts to extract all tryptic peptides in the input *de Bruijn* graph (Fig 2A), where a *tryptic peptide* is defined as a peptide encoded by a contiguous DNA sequence corresponding to a *path* spanning one or more edges in the graph that starts from a start codon (i.e., ATG) or after the codons encoding the trypsin cleavage sites (i.e., Lysine or Arginine), and ends before a stop codon or at the codons encoding trypsin cleavage sites. To accomplish this goal, the Graph2Pep algorithm adopts a depth-first search (DFS) strategy, starting from every start codon in each edge connecting to a source vertex, and traversing the graph until a desirable codon (i.e., a stop codon or a codon encoding a trypsin cleavage site) is encountered. The traversal continues from the next codon in the same reading frame until a trypsin cleavage site is encountered. On the other hand, when a stop codon is encountered, the traveral re-starts from a start codon in the same reading frame of the same edge where it terminates. Note that the algorithm enumerates the start codons in all three reading frames in every edge, but will not go through those in the reverse complementary strand because the reverse complementary strands are represented by different edges in the graph. Because the lengths of the edges in a *de Bruijn* graph from metagenome assembly can be substantially different, ranging from a few bases to hundred thousands of bases, to further accelerate the search, the Graph2Pep algorithm first predicts the protein-coding genes in all edges longer than a threshold (e.g., 500 bps by default) by using FragGeneScan, and includes the tryptic peptides in these proteins into the final results. After that, the depth-first search concentrates on the tryptic peptides encoded by DNA sequences on the short edges, in particular those encoded by DNA sequences spanning multiple short edges, and the search is automatically terminated when an edge longer than the threshold is encountered.

#### The Graph2Pro algorithm

Our second algorithm Graph2Pro is to further predict protein sequences from *de Bruijn* graph of metagenome assembly, using identified peptides as constraints. Given a *de Bruijn* graph assembly, and a set of identified (tryptic) peptides each mapped to a DNA segment (spanning one or more edges) in the graph, Graph2Pro attempts to retrieve a minimum set of protein sequences, each encoded by a series of codons starting from a start codon and ending in front of a stop codon, such that every identified peptide is contained in at least one protein. The Graph2Pro algorithm adopts the similar depth-first search strategy to traverse the *de Bruijn* graph, but will only traverse the subset of edges, in which each edge is spanned by the DNA segment of at least one identified peptide (Fig 2B). The predicted peptide sequences by Graph2Pro will compose a final target protein database subject to peptide identification in the metaproteomic data by using a search engine (i.e., MSGF+).

### Implementation and benchmarking experiments

We implemented the Graph2Pep and Graph2Pro algorithms in C++ and incorporated them into a pipeline for metaprotomics data analysis. We also included in our pipeline open source software tools (e.g., FragGeneScan and MSGF+) released by us and others previously, and several wrapper scripts in Python. These programs have been assembled in a streamline, and thus can be conveniently used for peptide/protein identification in metaproteomics when matched metagenomic and/or metatranscriptomic data are available. The package is available as open source software at https://github.com/COL-IU/Graph2Pro. In this study, we only consider the fully tryptic peptides in Graph2Pep algorithm. However, the program has one parameter allowing for adjusting the maximum number of mis-cleavages (default = 0). Note that it takes longer time to run the Graph2Pep program when mis-cleavages are allowed. In addition, the users can adjust another parameter of length threshold (default = 6 as used in this study) in the Graph2Pep program to filter peptides shorter than the threshold to be used in the first round of database searching. In a test case, the de Bruijn graph contains 18,523,653 edges and 37,047,308 vertices, from which 44,798,054 putative tryptic peptides are generated by Graph2Pep. The programs runs in 11 minutes and 22 seconds on a single CPU of Intel(R) Xeon(R) E5-2670 0 @ 2.60GHz.

#### Meta-omics data

We tested our tools and the pipeline using the experimental data from a recently published multi-omic study of oleaginous mixed microbial communities (OMMC) sampled from an anoxic biological wastewater treatment tank [24]. In particular, the metagenomic, metatranscriptomic and metaproteomic data were acquired from the community at four sample dates, denoted as SD3 (January 25th, 2011), SD5 (October 5th 2011), SD6 (October 12th, 2011) and SD7 (January 11th, 2012), respectively, in which SD3 and SD7 represent replications with similar physico-chemical conditions (e.g., temperatures), while SD5 and SD6 represent the control replications for the study of the microbial community in the sample SD3. As the metagenomic sequences of the sample SD5 yielded a poor coverage of the metagenome [24], we did not use the SD5 dataset in our benchmarking experiment and focused on the remaining three datasets (SD3, SD6 and SD7). For each of these datasets, the metagenomic (MG) and metatranscriptomic (MT) sequences were acquired by using the Illumina Genome Analyser (GA) IIx sequencers, resulting in paired-end reads. Metaproteomic samples were first processed by 1D-SDS-PAGE and in-gel reduction, prior to the alkylation and tryptic digestion. The resulting peptides were separated and analyzed by liquid chromatography (LC) coupled with tandem mass spectrometry (LC-MS/MS) by using LTQ-Orbitrap Elite (Thermo Fisher Scientific). We downloaded the metagenomic and metatranscriptomic datasets from the SRA website (SD3-MG: SRR1046369; SD3-MT: SRR1046681; SD6-MG: SRR1544596; SD6-MT:SRR1544599; SD7-MG: SRR1611146; and SD8-MT: SRR1611147). Raw reads were preprocessed using Trimmomatic (version 0.32) [44] and only reads of at least 80 bps were used in downstream analyses. We downloaded the spectra data from PeptideAtlas [45]: SD3 (ID: PASS00359), SD6 (PASS00577) and SD7 (PASS00578).

#### Metagenomic/metatranscriptomic assembly

We use SOAPdenovo2 [36] for the combined assembly of metagenomic and metatranscriptomic sequences. We selected the default parameters of SOAPdenovo2, and the k-mer size of 31. We have shown in our previous study that k-mer size of 31 is useful for maintaining the structure of assembly graph for later exploitation; when k-mer size gets too large, the graph becomes fragmented [43].

#### Peptide identification

We use MSGF+ [29] for peptide identification from a given protein sequence database. The parameters for the MSGF+ database searching is as the following: 1) instrument type: high-resolution LTQ; 2) precursor mass tolerance: 15ppm; 3) isotope error range: -1,2; 4) modifications: oxidation as variable and carboamidomethy as fixed; 5) maximum charge: 7; and 6) minimum charge: 1. The false discovery rate (FDR) is estimated by using a target-decoy search approach [46]. If the database consists of full length proteins predicted from FragGeneScan or Graph2Pro, we use the reverse protein sequences as decoy. If the database consists of peptides predicted from Graph2Pep, the decoy peptides were then generated by reversing the peptide sequences while preserving the C-terminal residues (K/R).

#### Functional annotation of identified proteins

We further predicted putative functions for identified proteins using similarity search based approaches. We used EggNOG database [47], which is the database of orthologous protein groups with annotated functions for functional annotation. Specifically, we searched identified proteins against the EggNOG protein database consisting of 14,875,530 protein sequences in 190,648 annotated COG (Cluster of Orthologous Groups) families by using RAPSearch2 [48] with its default parameter settings. A query protein is considered to hit a COG protein family if there is at least one protein in the family whose sequence alignment with the query protein has the sequence identity above 60% and e-value ≤ 10^−4^.

We also predicted putative pathways involving identified proteins as follows. First, the proteins were searched against the 90% non-redundant set of UniProt sequences (uniref90, downloaded from the Uniprot ftp website at ftp://ftp.uniprot.org) by RAPSearch2 [48]. Similarity search results were then used to predict potential enzymes (with EC assignments), which were further used to infer MetaCyc metabolic pathways by using MinPath [49]. MinPath takes the EC assignments as the input (and the EC to pathway mapping file ec2path, prepared based on the MetaCyc files pathways.dat and reactions.dat available at http://metacyc.org/download.shtml) and identifies the list of metabolic pathways that are needed to cover all annotated enzymes.

## Results

We implemented our graph-centric algorithms Graph2Pep and Graph2Pro in C++, and incorporated them into a pipeline for protein identification from metaproteomic MS/MS spectra data. We applied our pipeline to the waste water microbiome data, and the results show that our pipeline can significantly improve the identification of proteins from MS/MS spectra. Detailed information of identified proteins and their functional annotations are available in the supplementary data.

### Summary statistics of sequence assemblies

For each sample (SD3, SD6 and SD7), we assembled the combined datasets of metagenomic and metatranscriptomic sequences. The statistics of the assembly results and the protein-coding genes predicted from the contigs in the assemblies are summarized in Table 1. There are 19,553 contigs from the assembly of SD3 dataset with the N50 contig length of 840 bps, while more and longer contigs are assembled in SD6 and SD7 datasets. FragGeneScan predicted 32,760 protein-coding genes in the SD3 dataset, 113,135 genes for SD6 and 111,849 genes for SD7. Based on the graph structures of the assemblies, Graph2Pep output ∼ 16 million, ∼ 35 million and ∼ 33 million peptides in SD3, SD6 and SD7 datasets, respectively.

**Table 1:** Summary of the assemblies for three data sets used in the benchmarking experiments.

	SD3	SD6	SD7
No. of contigs	19,553	61,978	62,831
N50	840	934	938
No. of predicted genes	32,760	113,135	111,849
No. of predicted peptides (by Graph2Pep)	16,985,304	35,405,606	33,016,460

### Using assembly graph dramatically improves the protein identification

The assembly results (both the contigs and the assembly graph) of the combined metagenomic and metatranscriptomic datasets were used to predict peptides/proteins for MS/MS spectra identification. 603,867, 150,216, 148,310 MS/MS spectra in the samples of SD3, SD6 and SD7, respectively, were given as the input to the database search by MSGF+. The peptide identification results at the false-discovery rate of 1% are summarized in Table 2. We also showed MS/MS spectra identification based on proteins predicted from contigs for comparison. In SD3 dataset, we identified 18,498 spectra (PSMs, or peptide spectrum matches) using proteins predicted from contigs (by FragGeneScan), and 43,946 PSMs using peptides predicted from the assembly graph (by Graph2Pep) both at 1% FDR. In total, the first round of database searching identifies 13,928 unique peptides from 52,498 spectra, including 2,354 unique peptides and 9,496 spectra identified in both sets of FragGeneScan-predicted proteins and Graph2Pep-predicted peptides. The Venn diagrams of overlap between the identified unique peptides predicted by FragGeneScan and those predicted by Graph2Pep in the SD3, SD6 and SD7 datasets are shown in S1 Fig. Following the initial database searching, the identified unique peptides were mapped back to the assembly graph using Graph2Pro, and a total of 14,174 proteins were retrieved covering all identified peptides. To be noted here, in this step, we used the peptides of 5% FDR in order to increase the coverage of potential proteins in the sample.

**Table 2:** Summary of peptide identification in wastewater datasets based on the assembly of combined metagenomic and metatranscriptomic data.

	SD3		SD6		SD7	
	PSMs (%)	Unique Pep	PSMs (%)	Unique Pep	PSMs (%)	Unique Pep
FragGeneScan (i.e., using contigs only)	18,498 (3.06%)	4,736	9,055 (6.03%)	4,607	6,524 (4.40%)	3,540
Graph2Pep (1st)	43,946 (7.28%)	11,546	14,514 (9.66%)	7,528	16,761 (11.30%)	8,488
Union (1st)	52,498 (8.69%)	13,928	18,468 (12.29%)	9,743	19,184 (12.94%)	10,002
Graph2Pro (2nd)	**73,527 (12.18%)**	**18,162**	**23,849 (15.88%)**	**11,617**	**23,750 (16.01%)**	**11,366**

Proteins generated by Graph2Pro were then used as the new target database for a second round of peptide identification using MSGF+ on the same set of MS/MS spectra in the SD3 dataset, which identified a total of 18,162 unique peptides from 73,527 PSMs, corresponding to 12.18% of the whole input set of MS/MS spectra at 1% FDR. Comparing to the conventional protein identification procedure that identified 3.06% of MS/MS spectra from the proteins predicted in the contigs, the proposed pipeline identified about four times (398%) PSMs and unique peptides (383%). In particular, the second round of database search identified 21,029 (40.06%) more PSMs and 4,234 (30.40%) more unique peptides comparing with the first round of search, indicating the second traversal of the *de Bruijn* graph substantially increased the coverage of the target metaproteome. Similar levels of improvement were achieved on the other two datasets (252% and 321% for SD6 and SD7 datasets, respectively).

Our results showed that using assembly graphs of metagenome also significantly improved the identification of proteins from MS/MS spectra (Table 3). We take SD3 dataset as an example. A total of 2,043 proteins (that contains one or more identified peptides) can be identified using only the contigs. Out of 2,043 proteins, there are 1,065 proteins with at least two identified peptides. We note this number is comparable to the original results reported in Muller et al. [24], which reported 1,815 identified proteins. By contrast, 13,431 proteins can be identified when the assembly graph is used, while 3,245 proteins have at least two identified peptides. We clustered the combined set of 15,474 protein sequences based on a similarity cutoff of 0.8 by using CD-HIT [50], resulting in 11,209 clusters. Only 290 out of these 10,996 clusters contain proteins identified without using assembly graph, while 9,338 protein clusters contain only proteins identified by using using the assembly graph (and thus is rescued by the graph-centric approach). Similar results were obtained on the other two datasets (SD6 and SD7).

**Table 3:** Improvement of protein identification by using assembly graph.

	SD3	SD6	SD7
Muller et al^a^	1,815	-	-
#Proteins identified using contigs only	2,043	3,385	2,578
#Proteins identified using contigs only with at least two peptides	1,065	1,285	881
#Proteins identified using assembly graph	13,431	9,657	9,761
#Proteins identified using assembly graph with at least two peptides	3,245	2,340	2,164
#Clusters of proteins (-c 0.8)	11,209	7,928	7,926
#Clusters of proteins identified without using assembly graph	290	708	519
#Clusters of proteins rescued by using assembly graph	**9,338**	**5,114**	**5,663**

^a:^ the number of identified proteins was reported only for the SD3 sample in the paper [24].

### Improved protein identification leads to a better functional profiling of the microbial communities

We studied the impact of the expanded set of identified proteins by the graph-centric approaches on the downstream analysis. We focused on the functional categories of identified proteins and the metabolic pathways they are involved in.

**Fig 3:**
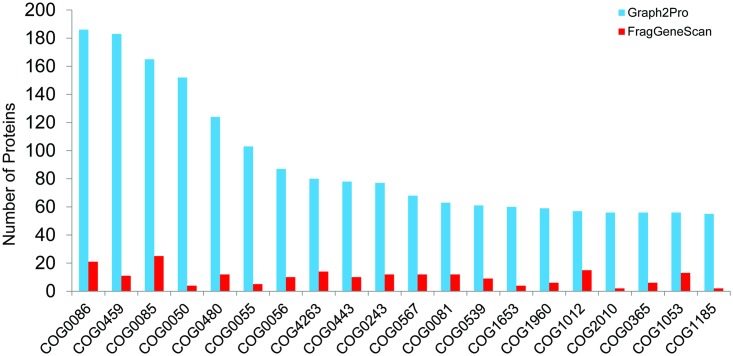
Comparison of the numbers of proteins in top 20 eggNOG families receiving the most hits of proteins identified in the SD3 sample by the graph-centric approach (Graph2Pro, blue) and the conventional approach (FragGeneScan, red).

Our graph-centric approaches enabled the identification of more proteins from the MS/MS spectra, revealing a more comprehensive functional profile of the microbial communities (with more eggNOG families identified). For the SD3 datasets, 8,706 out of 13,431 (64.82%) proteins in our expanded collection of identified proteins share similarity with eggNOG proteins, resulting in the identification of 1,206 COG families. By contrast, only 626 COG families were predicted using the 2,043 proteins identified by MS/MS spectra search against predicted proteins from contigs only (1,555 proteins share sequence similarities with eggNOG proteins). Table 4 lists the additional families predicted from our expanded collection of identified proteins, each supported by at least 10 proteins, and their annotations. Fig 3 shows the numbers of proteins in the top 20 eggNOG families with most proteins identified. Clearly, each of the functional categories is supported by considerably more proteins identified by the graph-centric approaches. We also conducted the functional analysis for the other two datasets (SD6 and SD7) and observed similar results (see S1 and S2 Tables and S2 and S3 Figs for details).

**Table 4:** The additional EggNOGs protein families identified with at least 10 protein hits by the graph-centric method.

COG ID	Category	Annotation	No.
COG0834	E	(ABC) transporter	26
COG2224	C	Isocitrate lyase	24
COG0359	J	Binds to the 23S rRNA (By similarity)	24
ENOG410XPVG	S	hydroxylamine oxidase	20
COG0234	O	Binds to cpn60 in the presence of Mg-ATP and suppresses the ATPase activity of the latter (By similarity)	20
COG0605	P	Destroys radicals which are normally produced within the cells and which are toxic to biological systems (By similarity)	17
COG0724	S	Rna-binding protein	15
COG4213	G	(ABC) transporter	13
COG0227	J	50s ribosomal protein L28	10
COG0366	G	alpha amylase, catalytic	10
COG0195	K	Transcription elongation factor NusA	10

Next we show that a more comprehensive coverage of metabolic pathways can be achieved by using our extended collection of proteins identified from metaproteomics data. A total of 213, 203, 223 MetaCyc metabolic pathways were reconstructured from SD3, SD6 and SD7 datasets, respectively, when proteins predicted from contigs only were used for MS/MS spectra identification. These numbers were increased to 328, 262, 294, respectively, when additional proteins were identified by our graph-centric approaches. In addition, our expanded collection of identified proteins provide a higher coverage of the pathways. Below we show two interesting pathways to demonstrate the importance of improved protein identification.

**Fig 4:**
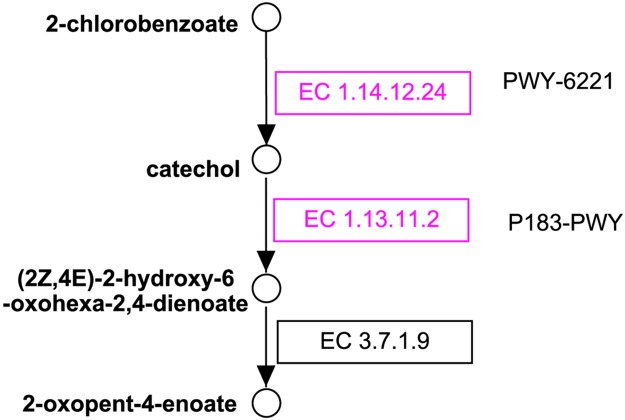
The 2-chlorobenzoate degradation pathway. Circles represent compounds, and boxes (with EC numbers) represent enzymes. Enzymes with MS/MS data support are highlighted in purple. The figure was prepared using PathVisio [54] based on the MetaCyc’s diagrams of pathways PWY-6221 and P183-PWY.

Table 5 shows the number of enzymes we identified in the wastewater datasets that are involved in the Rubisco shunt pathway (MetaCyc ID: PWY-5723; see the diagram at http://metacyc.org/META/NEW-IMAGE?type=NIL&object=PWY-5723). The results suggest that using assembly graph helps to increase the coverage of the pathway across all three datasets, SD3, SD6 and SD7. Rubisco shunt was first found in developing embryos of *Brassica napus* L. (oilseed rape), in which Rubisco (ribulose 1,5-bisphosphate carboxylase/oxygenase) acts without the Calvin cycle and increases the efficiency of carbon, resulting in 20% more acetyl-CoA and 40% less loss of carbon as CO2 [51]. We found MS/MS data supporting eight out of the nine enzymes involved in the Rubisco shunt. The eight enzymes we identified are EC.2.2.1.1, EC.2.2.1.2, EC.2.7.1.19, EC.2.7.1.40, EC.4.1.1.39 (Rubisco), EC.4.2.1.11, EC.5.1.3.1, and EC.5.3.1.6. For example, we identified a putative Rubisco in the SD3 dataset. The protein (Sequence ID: Protein12587; see the sequence in the FASTA file SD3.hybrid.fgsdbgraph.protein.fasta available at our website) contains 186 amino acids, which shares 94% sequence identity with a putative Rubisco identified in an uncultured bacterium (Sequence ID: gb∣AIF32007.1) according to the NCBI BLAST search. Strikingly, only three out of these enzymes were identified in the SD3 dataset when only the contigs were used (see Table 5). The second example (Fig 4) involves 2-chlorobenzoate degradation pathway (MetaCyc ID: PWY-6221) and catechol degradation to 2-oxopent-4-enoate I pathway (MetaCyc ID: P183-PWY). Enzymes involved in the degration of 2-chlorobenzoate degradation were detected persistently in all SD3, SD6 and SD7 samples. Chlorobenzoates are a group of compounds that occur in the environment either because of their release as herbicides or as products of bacterial degradation of polychlorinated biphenyls (PCBs; classified as a persistent organic pollutant, due to their environmental toxicity [52]). The reaction that converts 2-chlorobenzoate to catechol was first identified in *Burkholderia cepacia* 2CBS, which was shown to be able to grow with 2-chlorobenzoate as the sole source for carbon and energy [53]. Two key functions involved in the 2-chlorobenzoate degradation, i.e., EC.1.14.12.24 and EC.1.13.11.2, were supported by identified proteins in our collection (highlighted in purple in Fig 4; both enzymes were identified in SD3 dataset by our approach, but none were identified if only contigs were used to predict reference genes; and in SD7 dataset, EC.1.13.11.2 was rescued by using assembly graph).

**Table 5:** The number of identified enzymes involved in the Rubisco shunt.

	SD3	SD6	SD7
Contigs only	3	6	5
Graph2Pro	8	7	6

## Discussion

In this paper, we presented two algorithms (Graph2Pep and Graph2Pro) for metaproteomic data analysis based on a graph-centric approach, in which the *de Bruijn* graph representation of the assembly of metagenomic sequences (or of the combined set of metagenomic and metatranscriptomic sequences) is used to produce the target protein database subject to the protein identification using metaproteomic data. We tested the algorithms on the metaproteomic datasets from a wastewater study in which matched metagenomic and metatranscriptomic data were also acquired. Comparing with the conventional method where the target protein database was constructed from the proteins predicted from the assembly contigs, our graph-centric approach significantly improved the protein identifications. Notably, although in this study, we consider the trypsin as the digestion enzyme, which is used by most metaproteomics projects, our algorithms can handle data collected by using other digestion enzymes, where the users need to define a different set of amino acid residues as the cleavage sites (e.g., the glutamyl and aspartyl residues when Glu-C is used) in our programs. We also note that more proteins can be identified when the assembly of combined metagenomic and metatranscriptomic datasets is used, when both metagenomic and metatranscriptomic datasets are available.

The graph-centric approach presented here relies on the *de Bruijn* graph representation of the sequence assembly (either from metagenomic sequences or from the combined metagenomic and metatranscriptomic sequences). In our pipeline, we utilized the output of SOAPdenovo that contains the topology of the *de Bruijn* graph in addition to the contig sequences (each corresponding to an edge in the graph). Many other metagenome assembly algorithms (e.g., metaVelvet [55] and meta-IDBA [56]) are based on the data structure of *de Bruijn* graph, which, however do not output the graph structure explicitly. As a successor of SOAPdenovo for metagenome assembly, the MegaHIT algorithm [37] can output the *de Bruijn* graph topology as temporary files in FASTG format, which is designed to incorporate allelic polymorphism and assembly uncertainty in an assembly graph [57]. The recently released metaSPAdes assembler [42] adapted the core SPAdes algorithm for metagenome assembly, and also output the assembly graph in FASTG format. Our current algorithms of Graph2Pep and Graph2Pro can support the input assembly graph in FASTG format, but has not been tested for its performance using the output from the other metagenome assemblers. Here, we would like to encourage the *de Bruijn* graph based assembly algorithms to allow users to generate explicit output of *de Bruijn* graphs (e.g., in FASTG format) that will be valuable for downstream analysis (such as the metatranscriptomic and metaproteomic analysis guided by metagenome assembly, as presented here).

Our graph-centric approaches are shown to be effective for improving the protein identification from metaproteomic MS/MS data. However, considering the fact that complex microbial communities contain hundreds or even thousands of species with highly uneven abundances, it will be both experimentally and computationally challenging to detect all proteins produced by the species, especially the proteins produced by the rare species in the community.

The ultimate goal of metaproteomics is not only to identify proteins expressed in the microbial community, but also to estimate their abundances (i.e., their expression levels) under different conditions. Nevertheless, a protein can be quantified only if it can be identified by using the metaproteomic data. Therefore, the methods presented here that increase the coverage of protein identification will also help the subsequent steps for protein quantification. We plan to implement the functionality of protein quantification based on label-free quantification approaches in the future release of our software.

## Supporting Information

S1 Table The additional COG families identified in SD6 dataset. (PDF)

S2 Table The additional COG families identified in SD7 dataset. (PDF)

S1 Fig The overlap of unique peptides between FragGeneScan and Graph2Pep in dataset SD3,SD6 and SD7 datasets. (JPG)

S2 Fig The comparison of identified protein in COG families in SD6 dataset. (JPG)

S3 Fig The comparison of identified protein in COG families in SD7 dataset. (JPG)

S1 Dataset The detailed peptides/proteins identification and functional analysis of identified proteins. (ZIP)
