# Supplementary material for: A Graph-Centric Approach for Metagenome-Guided Peptide and Protein Identification in Metaproteomics
Source: PLoS Comput Biol. 2016 Dec 5;12(12):e1005224. doi: 10.1371/journal.pcbi.1005224 (PMC5137872; doi:10.1371/journal.pcbi.1005224)
Supplement: S1 Table — (PDF) [file pcbi.1005224.s001.pdf]

COG ID	Category	Annotation	No.
COG3497	S	tail sheath protein	9
COG2036	B	Core component of nucleosome. Nucleosomes wrap and compact DNA into chromatin, limiting DNA accessibility to the cellular machineries which require DNA as a template. Histones thereby play a central role in transcription regulation, DNA repair, DNA replication and chromosomal stability. DNA accessibility is regulated via a complex set of post-translational modifications of histones, also called histone code, and nucleosome remodeling	8
COG1152	C	Part of a complex that catalyzes the reversible cleavage of acetyl-CoA, allowing autotrophic growth from CO(2) (By similarity)	8
ENOG410Y0ZG	C	monooxygenase, subunit B	6
COG1456	C	Acetyl-CoA decarbonylase synthase complex subunit gamma	6
ENOG4111NV5	B	Core component of nucleosome. Nucleosomes wrap and compact DNA into chromatin, limiting DNA accessibility to the cellular machineries which require DNA as a template. Histones thereby play a central role in transcription regulation, DNA repair, DNA replication and chromosomal stability. DNA accessibility is regulated via a complex set of post-translational modifications of histones, also called histone code, and nucleosome remodeling	6
COG4770	I	carboxylase	5
COG0488	S	(ABC) transporter	5
ENOG41125TR	S	dna-binding protein	5
COG1156	C	Produces ATP from ADP in the presence of a proton gradient across the membrane. The V-type beta chain is a regulatory subunit (By similarity)	5
