# Supplementary material for: A Graph-Centric Approach for Metagenome-Guided Peptide and Protein Identification in Metaproteomics
Source: PLoS Comput Biol. 2016 Dec 5;12(12):e1005224. doi: 10.1371/journal.pcbi.1005224 (PMC5137872; doi:10.1371/journal.pcbi.1005224)
Supplement: S2 Table — (PDF) [file pcbi.1005224.s002.pdf]

COG ID	Category	Annotation	No.
COG0049	J	One of the primary rRNA binding proteins, it binds directly to 16S rRNA where it nucleates assembly of the head domain of the 30S subunit. Is located at the subunit interface close to the decoding center, probably blocks exit of the E-site tRNA (By similarity)	22
COG0268	J	Binds directly to 16S ribosomal RNA (By similarity)	11
COG0076	E	decarboxylase	10
COG2414	C	aldehyde ferredoxin oxidoreductase	9
COG4969	U	Fimbrial protein	9
COG0244	J	50s ribosomal protein L10	9
COG2036	B	Core component of nucleosome. Nucleosomes wrap and compact DNA into chromatin, limiting DNA accessibility to the cellular machineries which require DNA as a template. Histones thereby play a central role in transcription regulation, DNA repair, DNA replication and chromosomal stability. DNA accessibility is regulated via a complex set of post-translational modifications of histones, also called histone code, and nucleosome remodeling	8
COG5531	B	SWI SNF related, matrix associated, actin dependent regulator of chromatin, subfamily d, member	8
COG5256	J	This protein promotes the GTP-dependent binding of aminoacyl-tRNA to the A-site of ribosomes during protein biosynthesis (By similarity)	8
ENOG410XPVG	S	hydroxylamine oxidase	7
