# Supplementary figures and images for: A Graph-Centric Approach for Metagenome-Guided Peptide and Protein Identification in Metaproteomics
Source: PLoS Comput Biol. 2016 Dec 5;12(12):e1005224. doi: 10.1371/journal.pcbi.1005224 (PMC5137872; doi:10.1371/journal.pcbi.1005224)

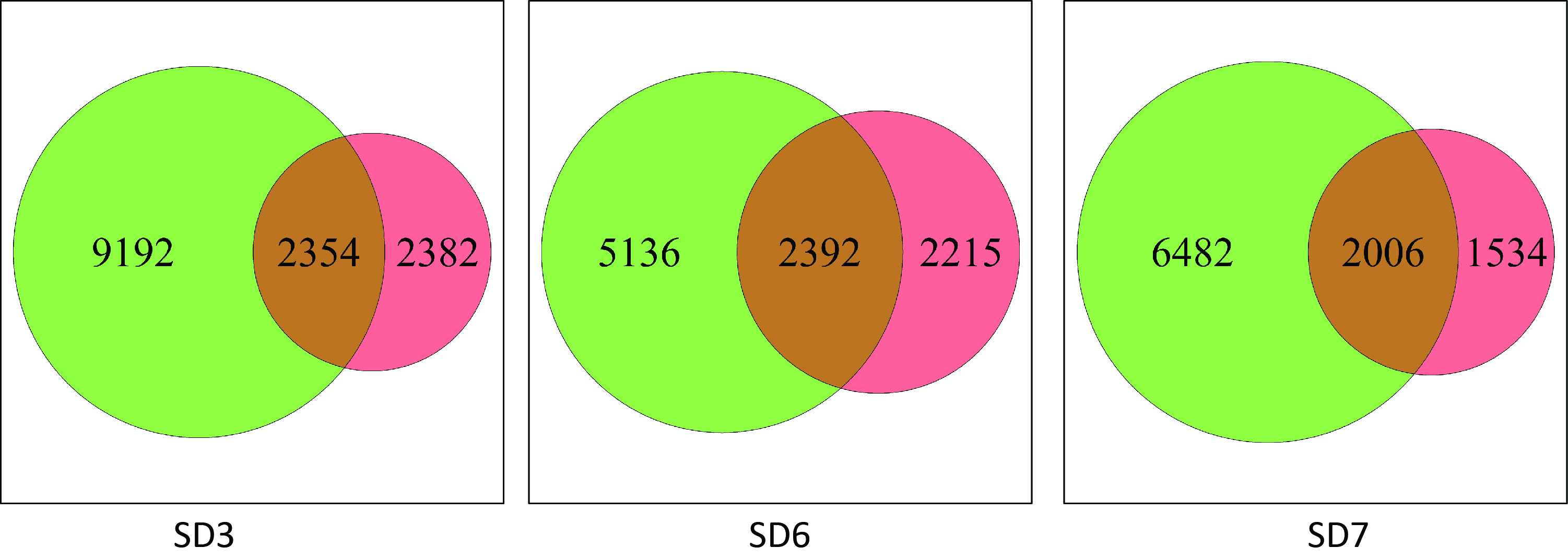

Supplement: S1 Fig — (JPG) [file pcbi.1005224.s003.jpg]

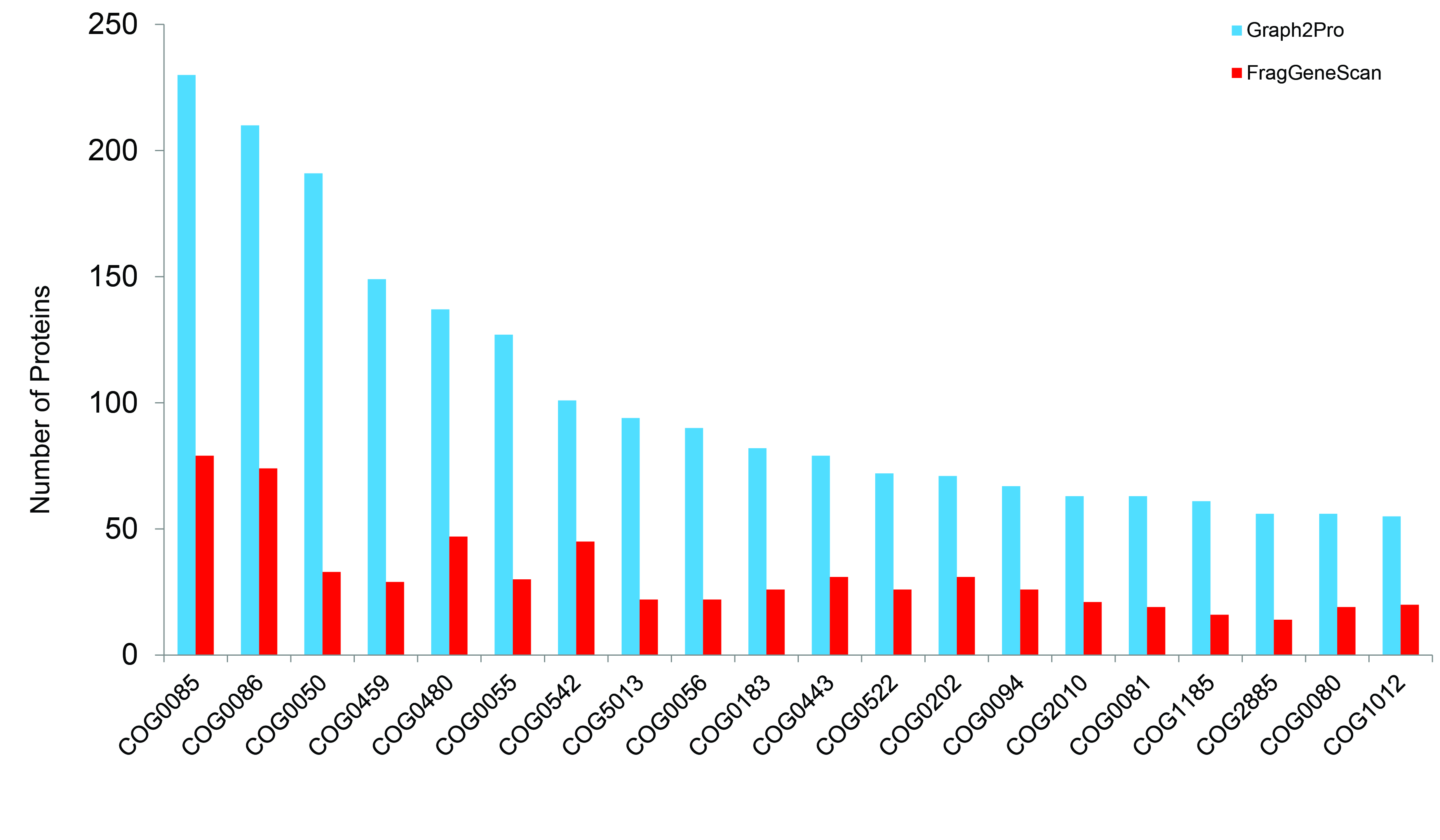

Supplement: S2 Fig — (JPG) [file pcbi.1005224.s004.jpg]

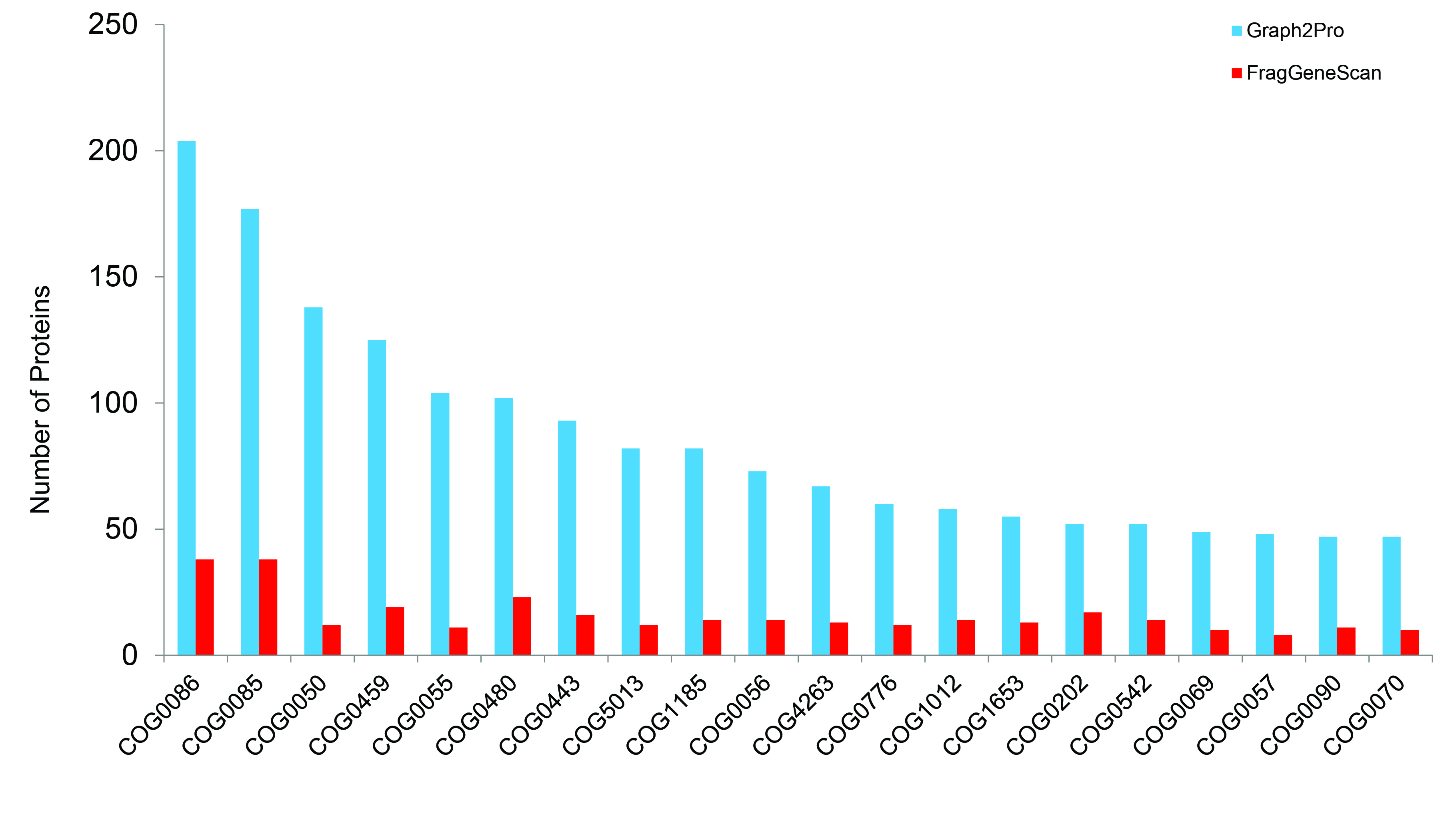

Supplement: S3 Fig — (JPG) [file pcbi.1005224.s005.jpg]
