# Supplementary material for: A Graph-Centric Approach for Metagenome-Guided Peptide and Protein Identification in Metaproteomics
Source: PLoS Comput Biol. 2016 Dec 5;12(12):e1005224. doi: 10.1371/journal.pcbi.1005224 (PMC5137872; doi:10.1371/journal.pcbi.1005224)
Supplement: S1 Dataset — (ZIP) [file pcbi.1005224.s006.zip › Supplement/Pathway/SD3.hybrid.fgsdbgraph-pwy.html]

### Metabolic pathway summary

| Pathway | Description | #Functions | #Annotated | Functions |
| --- | --- | --- | --- | --- |
| P105-PWY | TCA cycle IV (2-oxoglutarate decarboxylase) | 10 | 8 | EC.1.1.1.37 EC.1.1.1.42 EC.1.3.5.1 EC.2.3.3.1 EC.2.3.3.9 EC.4.1.1.71 EC.4.1.3.1 EC.4.2.1.2 |
| P108-PWY | pyruvate fermentation to propanoate I | 7 | 4 | EC.1.1.1.37 EC.4.2.1.2 EC.5.1.99.1 EC.5.4.99.2 |
| P122-PWY | heterolactic fermentation | 18 | 9 | EC.1.1.1.1 EC.1.2.1.12 EC.2.3.1.8 EC.2.7.1.40 EC.2.7.2.3 EC.3.1.1.31 EC.4.2.1.11 EC.5.1.3.1 EC.5.3.1.9 |
| P124-PWY | Bifidobacterium shunt | 15 | 9 | EC.1.2.1.12 EC.2.2.1.1 EC.2.2.1.2 EC.2.7.1.40 EC.2.7.2.3 EC.4.2.1.11 EC.5.1.3.1 EC.5.3.1.6 EC.5.3.1.9 |
| P142-PWY | pyruvate fermentation to acetate I | 1 | 1 | EC.1.2.7.1 |
| P164-PWY | purine nucleobases degradation I (anaerobic) | 12 | 2 | EC.1.17.1.4 EC.1.2.1.2 |
| P183-PWY | catechol degradation to 2-oxopent-4-enoate I | 2 | 1 | EC.1.13.11.2 |
| P184-PWY | protocatechuate degradation I (*meta*-cleavage pathway) | 8 | 2 | EC.4.1.1.3 EC.4.1.3.17 |
| P185-PWY | formaldehyde assimilation III (dihydroxyacetone cycle) | 11 | 9 | EC.1.2.1.12 EC.2.2.1.1 EC.2.2.1.2 EC.2.7.2.3 EC.3.1.3.11 EC.4.1.2.13 EC.5.1.3.1 EC.5.3.1.1 EC.5.3.1.6 |
| P21-PWY | pentose phosphate pathway (partial) | 2 | 2 | EC.2.2.1.1 EC.5.1.3.1 |
| P224-PWY | sulfate reduction V (dissimilatory) | 3 | 3 | EC.1.8.99.2 EC.1.8.99.3 EC.2.7.7.4 |
| P23-PWY | reductive TCA cycle I | 10 | 7 | EC.1.1.1.37 EC.1.1.1.42 EC.1.2.7.1 EC.1.2.7.3 EC.2.7.9.2 EC.4.2.1.2 EC.6.2.1.5 |
| P341-PWY | glycolysis V (Pyrococcus) | 9 | 5 | EC.2.7.1.40 EC.4.1.2.13 EC.4.2.1.11 EC.5.3.1.1 EC.5.3.1.9 |
| P42-PWY | incomplete reductive TCA cycle | 7 | 5 | EC.1.1.1.37 EC.1.2.7.1 EC.1.2.7.3 EC.4.2.1.2 EC.6.2.1.5 |
| P483-PWY | phosphonoacetate degradation | 1 | 1 | EC.3.11.1.2 |
| PEPTIDOGLYCANSYN-PWY | peptidoglycan biosynthesis I (*meso*-diaminopimelate containing) | 2 | 1 | EC.2.7.8.13 |
| PLPSAL-PWY | pyridoxal 5'-phosphate salvage I | 2 | 1 | EC.1.4.3.5 |
| PROPIONMET-PWY | propanoyl CoA degradation I | 3 | 3 | EC.5.1.99.1 EC.5.4.99.2 EC.6.4.1.3 |
| PROSYN-PWY | L-proline biosynthesis I | 3 | 2 | EC.1.2.1.41 EC.1.5.1.2 |
| PROTOCATECHUATE-ORTHO-CLEAVAGE-PWY | protocatechuate degradation II (ortho-cleavage pathway) | 4 | 1 | EC.1.13.11.3 |
| PWY-101 | photosynthesis light reactions | 4 | 1 | EC.1.18.1.2 |
| PWY-1042 | glycolysis IV (plant cytosol) | 10 | 8 | EC.1.2.1.12 EC.2.7.1.11 EC.2.7.1.40 EC.2.7.1.90 EC.2.7.2.3 EC.4.1.2.13 EC.4.2.1.11 EC.5.3.1.1 |
| PWY-1081 | homogalacturonan degradation | 2 | 1 | EC.3.1.1.11 |
| PWY-1281 | sulfoacetaldehyde degradation I | 2 | 1 | EC.2.3.1.8 |
| PWY-181 | photorespiration | 8 | 2 | EC.1.1.3.15 EC.2.1.2.1 |
| PWY-1861 | formaldehyde assimilation II (RuMP Cycle) | 8 | 6 | EC.2.2.1.1 EC.2.2.1.2 EC.2.7.1.11 EC.4.1.2.13 EC.5.1.3.1 EC.5.3.1.6 |
| PWY-1881 | formate oxidation to CO2 | 1 | 1 | EC.1.2.1.2 |
| PWY-2161 | folate polyglutamylation | 3 | 3 | EC.2.1.2.1 EC.6.3.2.17 EC.6.3.4.3 |
| PWY-2201 | folate transformations I | 11 | 4 | EC.1.5.1.20 EC.2.1.1.13 EC.2.1.2.1 EC.6.3.4.3 |
| PWY-2221 | Entner-Doudoroff pathway III (semi-phosphorylative) | 13 | 4 | EC.1.2.1.3 EC.2.7.1.40 EC.3.1.1.17 EC.4.2.1.11 |
| PWY-2301 | *myo*-inositol biosynthesis | 2 | 2 | EC.3.1.3.25 EC.5.5.1.4 |
| PWY-241 | C4 photosynthetic carbon assimilation cycle, NADP-ME type | 5 | 3 | EC.1.1.1.40 EC.2.7.9.1 EC.4.2.1.1 |
| PWY-2501 | fatty acid α-oxidation I | 2 | 1 | EC.1.2.1.3 |
| PWY-2503 | benzoate degradation I (aerobic) | 2 | 1 | EC.1.14.12.10 |
| PWY-2722 | trehalose degradation IV | 4 | 1 | EC.2.4.1.64 |
| PWY-2941 | L-lysine biosynthesis II | 8 | 4 | EC.1.17.1.8 EC.1.2.1.11 EC.2.7.2.4 EC.4.3.3.7 |
| PWY-2942 | L-lysine biosynthesis III | 7 | 5 | EC.1.17.1.8 EC.1.2.1.11 EC.1.4.1.16 EC.2.7.2.4 EC.4.3.3.7 |
| PWY-3121 | linamarin degradation | 2 | 1 | EC.3.2.1.21 |
| PWY-3161 | indole-3-acetate biosynthesis III (bacteria) | 2 | 1 | EC.3.5.1.4 |
| PWY-3221 | dTDP-L-rhamnose biosynthesis II | 2 | 2 | EC.2.7.7.24 EC.4.2.1.46 |
| PWY-3341 | L-proline biosynthesis III | 2 | 1 | EC.1.5.1.2 |
| PWY-3641 | L-carnitine degradation III | 4 | 1 | EC.1.1.1.39 |
| PWY-3722 | glycine betaine biosynthesis II (Gram-positive bacteria) | 2 | 1 | EC.1.2.1.8 |
| PWY-3781 | aerobic respiration I (cytochrome c) | 4 | 4 | EC.1.10.2.2 EC.1.3.5.1 EC.1.6.5.3 EC.1.9.3.1 |
| PWY-381 | nitrate reduction II (assimilatory) | 3 | 2 | EC.1.7.1.1 EC.6.3.1.2 |
| PWY-3841 | folate transformations II | 9 | 5 | EC.1.5.1.20 EC.1.5.1.3 EC.2.1.1.13 EC.2.1.2.1 EC.6.3.4.3 |
| PWY-40 | putrescine biosynthesis I | 2 | 1 | EC.4.1.1.19 |
| PWY-4041 | γ-glutamyl cycle | 4 | 1 | EC.3.5.2.9 |
| PWY-4061 | glutathione-mediated detoxification I | 5 | 1 | EC.2.5.1.18 |
| PWY-4081 | glutathione redox reactions I | 3 | 2 | EC.1.11.1.9 EC.1.8.1.7 |
| PWY-4261 | glycerol degradation I | 2 | 2 | EC.1.1.5.3 EC.2.7.1.30 |
| PWY-4302 | aerobic respiration III (alternative oxidase pathway) | 3 | 2 | EC.1.3.5.1 EC.1.6.5.3 |
| PWY-4321 | L-glutamate degradation IV | 5 | 1 | EC.4.1.1.15 |
| PWY-4341 | L-glutamate biosynthesis V | 1 | 1 | EC.1.4.7.1 |
| PWY-4381 | fatty acid biosynthesis initiation I | 5 | 2 | EC.2.3.1.85 EC.6.4.1.2 |
| PWY-4521 | arsenite oxidation I (respiratory) | 2 | 1 | EC.1.9.3.1 |
| PWY-46 | putrescine biosynthesis III | 1 | 1 | EC.4.1.1.17 |
| PWY-4722 | creatinine degradation II | 5 | 1 | EC.3.5.2.14 |
| PWY-4821 | UDP-D-xylose biosynthesis | 1 | 1 | EC.4.1.1.35 |
| PWY-4981 | L-proline biosynthesis II (from arginine) | 5 | 2 | EC.1.5.1.2 EC.2.1.3.3 |
| PWY-4983 | L-citrulline-nitric oxide cycle | 3 | 2 | EC.4.3.2.1 EC.6.3.4.5 |
| PWY-4984 | urea cycle | 5 | 3 | EC.2.1.3.3 EC.4.3.2.1 EC.6.3.4.5 |
| PWY-5 | canavanine biosynthesis | 3 | 2 | EC.4.3.2.1 EC.6.3.4.5 |
| PWY-5025 | indole-3-acetate biosynthesis IV (bacteria) | 2 | 1 | EC.3.5.1.4 |
| PWY-5028 | L-histidine degradation II | 5 | 2 | EC.4.2.1.49 EC.4.3.1.3 |
| PWY-5041 | *S*-adenosyl-L-methionine cycle II | 4 | 2 | EC.2.5.1.6 EC.3.3.1.1 |
| PWY-5057 | L-valine degradation II | 3 | 2 | EC.1.1.1.1 EC.2.6.1.42 |
| PWY-5076 | L-leucine degradation III | 4 | 2 | EC.1.1.1.1 EC.2.6.1.42 |
| PWY-5078 | L-isoleucine degradation II | 3 | 2 | EC.1.1.1.1 EC.2.6.1.42 |
| PWY-5079 | L-phenylalanine degradation III | 4 | 2 | EC.1.1.1.1 EC.2.6.1.57 |
| PWY-5082 | L-methionine degradation III | 2 | 1 | EC.1.1.1.1 |
| PWY-5084 | 2-oxoglutarate decarboxylation to succinyl-CoA | 3 | 3 | EC.1.2.4.2 EC.1.8.1.4 EC.2.3.1.61 |
| PWY-5097 | L-lysine biosynthesis VI | 7 | 4 | EC.1.17.1.8 EC.1.2.1.11 EC.2.7.2.4 EC.4.3.3.7 |
| PWY-5103 | L-isoleucine biosynthesis III | 5 | 4 | EC.1.1.1.86 EC.2.2.1.6 EC.2.6.1.42 EC.4.2.1.9 |
| PWY-5104 | L-isoleucine biosynthesis IV | 6 | 5 | EC.1.2.7.1 EC.2.2.1.6 EC.2.6.1.42 EC.4.2.1.9 EC.6.2.1.17 |
| PWY-5108 | L-isoleucine biosynthesis V | 2 | 1 | EC.2.6.1.42 |
| PWY-5136 | fatty acid β-oxidation II (peroxisome) | 5 | 4 | EC.1.1.1.35 EC.2.3.1.16 EC.4.2.1.17 EC.6.2.1.3 |
| PWY-5137 | fatty acid β-oxidation III (unsaturated, odd number) | 1 | 1 | EC.5.3.3.8 |
| PWY-5138 | unsaturated, even numbered fatty acid β-oxidation | 5 | 4 | EC.1.3.1.34 EC.4.2.1.17 EC.5.1.2.3 EC.5.3.3.8 |
| PWY-5143 | long-chain fatty acid activation | 1 | 1 | EC.6.2.1.3 |
| PWY-5154 | L-arginine biosynthesis III (via *N*-acetyl-L-citrulline) | 9 | 6 | EC.1.2.1.38 EC.2.3.1.1 EC.2.7.2.8 EC.4.3.2.1 EC.6.3.4.5 EC.6.3.5.5 |
| PWY-5177 | glutaryl-CoA degradation | 4 | 2 | EC.1.1.1.35 EC.2.3.1.9 |
| PWY-5188 | tetrapyrrole biosynthesis I (from glutamate) | 6 | 5 | EC.2.5.1.61 EC.4.2.1.24 EC.4.2.1.75 EC.5.4.3.8 EC.6.1.1.17 |
| PWY-5189 | tetrapyrrole biosynthesis II (from glycine) | 4 | 3 | EC.2.5.1.61 EC.4.2.1.24 EC.4.2.1.75 |
| PWY-5194 | siroheme biosynthesis | 2 | 1 | EC.1.3.1.76 |
| PWY-5265 | peptidoglycan biosynthesis II (staphylococci) | 4 | 3 | EC.2.4.1.129 EC.2.7.8.13 EC.3.4.16.4 |
| PWY-5278 | sulfite oxidation III | 2 | 2 | EC.1.8.99.2 EC.2.7.7.4 |
| PWY-5279 | sulfite oxidation II | 2 | 1 | EC.1.8.99.2 |
| PWY-5316 | nicotine biosynthesis | 5 | 1 | EC.2.4.2.19 |
| PWY-5340 | sulfate activation for sulfonation | 2 | 2 | EC.2.7.1.25 EC.2.7.7.4 |
| PWY-5344 | L-homocysteine biosynthesis | 2 | 1 | EC.2.5.1.49 |
| PWY-5372 | carbon tetrachloride degradation II | 2 | 1 | EC.1.2.99.2 |
| PWY-5392 | reductive TCA cycle II | 10 | 5 | EC.1.1.1.37 EC.1.2.7.1 EC.1.2.7.3 EC.4.2.1.2 EC.6.2.1.5 |
| PWY-5399 | betacyanin biosynthesis | 2 | 1 | EC.1.14.18.1 |
| PWY-5443 | aminopropanol phosphate biosynthesis I | 2 | 1 | EC.4.1.1.81 |
| PWY-5458 | methylglyoxal degradation V | 3 | 1 | EC.1.1.2.3 |
| PWY-5461 | betanidin degradation | 1 | 1 | EC.1.11.1.7 |
| PWY-5468 | lupanine biosynthesis | 2 | 1 | EC.4.1.1.18 |
| PWY-5480 | pyruvate fermentation to ethanol I | 3 | 2 | EC.1.1.1.1 EC.2.3.1.54 |
| PWY-5483 | pyruvate fermentation to acetate III | 2 | 1 | EC.1.2.7.1 |
| PWY-5484 | glycolysis II (from fructose 6-phosphate) | 11 | 9 | EC.1.2.1.12 EC.2.7.1.11 EC.2.7.1.40 EC.2.7.2.3 EC.2.7.9.2 EC.3.1.3.11 EC.4.1.2.13 EC.4.2.1.11 EC.5.3.1.1 |
| PWY-5485 | pyruvate fermentation to acetate IV | 3 | 2 | EC.2.3.1.54 EC.2.3.1.8 |
| PWY-5486 | pyruvate fermentation to ethanol II | 2 | 1 | EC.1.1.1.1 |
| PWY-5491 | diethylphosphate degradation | 2 | 1 | EC.3.1.3.1 |
| PWY-5493 | reductive monocarboxylic acid cycle | 2 | 2 | EC.1.2.7.1 EC.2.3.1.54 |
| PWY-5531 | chlorophyllide *a* biosynthesis II (anaerobic) | 7 | 3 | EC.1.3.3.3 EC.4.1.1.37 EC.6.6.1.1 |
| PWY-5533 | acetone degradation II (to acetoacetate) | 2 | 1 | EC.6.4.1.6 |
| PWY-5537 | pyruvate fermentation to acetate V | 2 | 1 | EC.6.2.1.5 |
| PWY-5538 | pyruvate fermentation to acetate VI | 2 | 2 | EC.1.2.7.1 EC.6.2.1.5 |
| PWY-5600 | pyruvate fermentation to acetate VII | 1 | 1 | EC.1.2.7.1 |
| PWY-561 | superpathway of glyoxylate cycle and fatty acid degradation | 4 | 4 | EC.1.1.1.37 EC.1.3.5.1 EC.4.1.1.49 EC.4.2.1.2 |
| PWY-5642 | 2,4-dinitrotoluene degradation | 3 | 1 | EC.1.2.1.27 |
| PWY-5647 | 2-nitrobenzoate degradation I | 5 | 1 | EC.4.1.1.45 |
| PWY-5651 | L-tryptophan degradation to 2-amino-3-carboxymuconate semialdehyde | 6 | 1 | EC.3.5.1.9 |
| PWY-5652 | 2-amino-3-carboxymuconate semialdehyde degradation to glutaryl-CoA | 2 | 1 | EC.4.1.1.45 |
| PWY-5659 | GDP-mannose biosynthesis | 4 | 3 | EC.5.3.1.8 EC.5.3.1.9 EC.5.4.2.8 |
| PWY-5667 | CDP-diacylglycerol biosynthesis I | 4 | 2 | EC.1.1.1.94 EC.2.3.1.51 |
| PWY-5676 | acetyl-CoA fermentation to butanoate II | 6 | 3 | EC.1.1.1.36 EC.1.3.8.1 EC.2.3.1.9 |
| PWY-5686 | UMP biosynthesis | 6 | 3 | EC.2.1.3.2 EC.2.4.2.10 EC.6.3.5.5 |
| PWY-5690 | TCA cycle II (plants and fungi) | 7 | 5 | EC.1.1.1.37 EC.1.3.5.1 EC.2.3.3.1 EC.4.2.1.2 EC.6.2.1.5 |
| PWY-5691 | urate degradation to allantoin I | 3 | 1 | EC.1.7.3.3 |
| PWY-5695 | urate biosynthesis/inosine 5'-phosphate degradation | 4 | 2 | EC.1.1.1.205 EC.1.17.1.4 |
| PWY-5704 | urea degradation II | 1 | 1 | EC.3.5.1.5 |
| PWY-5723 | Rubisco shunt | 9 | 8 | EC.2.2.1.1 EC.2.2.1.2 EC.2.7.1.19 EC.2.7.1.40 EC.4.1.1.39 EC.4.2.1.11 EC.5.1.3.1 EC.5.3.1.6 |
| PWY-5739 | GDP-D-perosamine biosynthesis | 2 | 1 | EC.4.2.1.47 |
| PWY-5747 | 2-methylcitrate cycle II | 5 | 2 | EC.4.2.1.99 EC.6.2.1.17 |
| PWY-5754 | 4-hydroxybenzoate biosynthesis I (eukaryotes) | 5 | 2 | EC.2.6.1.57 EC.3.1.2.23 |
| PWY-5791 | 1,4-dihydroxy-2-naphthoate biosynthesis II (plants) | 7 | 2 | EC.4.1.3.36 EC.6.2.1.26 |
| PWY-5886 | 4-hydroxyphenylpyruvate biosynthesis | 2 | 1 | EC.2.6.1.57 |
| PWY-5913 | TCA cycle VI (obligate autotrophs) | 10 | 6 | EC.1.1.1.37 EC.1.1.1.42 EC.2.3.3.1 EC.2.6.1.1 EC.4.2.1.2 EC.6.2.1.5 |
| PWY-5921 | glutaminyl-tRNAgln biosynthesis via transamidation | 1 | 1 | EC.6.3.5.7 |
| PWY-5938 | (*R*)-acetoin biosynthesis I | 2 | 1 | EC.2.2.1.6 |
| PWY-5939 | (*R*)-acetoin biosynthesis II | 2 | 1 | EC.2.2.1.6 |
| PWY-5941 | glycogen degradation II (eukaryotic) | 6 | 2 | EC.2.4.1.1 EC.5.4.2.2 |
| PWY-5958 | acridone alkaloid biosynthesis | 4 | 1 | EC.4.1.3.27 |
| PWY-5971 | palmitate biosynthesis II (bacteria and plants) | 9 | 5 | EC.1.1.1.100 EC.1.3.1.9 EC.2.3.1.85 EC.4.2.1.59 EC.6.2.1.3 |
| PWY-5973 | *cis*-vaccenate biosynthesis | 5 | 4 | EC.1.1.1.100 EC.1.3.1.9 EC.2.3.1.179 EC.4.2.1.59 |
| PWY-5981 | CDP-diacylglycerol biosynthesis III | 4 | 2 | EC.1.1.1.94 EC.2.3.1.51 |
| PWY-5989 | stearate biosynthesis II (bacteria and plants) | 7 | 4 | EC.1.1.1.100 EC.1.3.1.9 EC.4.2.1.59 EC.6.2.1.3 |
| PWY-6000 | γ-linolenate biosynthesis II (animals) | 2 | 2 | EC.1.14.19.3 EC.6.2.1.3 |
| PWY-6001 | linoleate biosynthesis II (animals) | 2 | 1 | EC.6.2.1.3 |
| PWY-6002 | lotaustralin degradation | 2 | 1 | EC.3.2.1.21 |
| PWY-6054 | dimethylsulfoniopropanoate biosynthesis I (Wollastonia) | 2 | 1 | EC.1.2.1.3 |
| PWY-6055 | dimethylsulfoniopropanoate biosynthesis II (Spartina) | 2 | 1 | EC.1.2.1.3 |
| PWY-6061 | bile acid biosynthesis, neutral pathway | 12 | 2 | EC.2.3.1.176 EC.5.1.99.4 |
| PWY-6113 | superpathway of mycolate biosynthesis | 4 | 3 | EC.1.1.1.100 EC.1.3.1.9 EC.4.2.1.59 |
| PWY-6118 | glycerol-3-phosphate shuttle | 2 | 1 | EC.1.1.5.3 |
| PWY-6121 | 5-aminoimidazole ribonucleotide biosynthesis I | 5 | 2 | EC.2.4.2.14 EC.6.3.5.3 |
| PWY-6123 | inosine-5'-phosphate biosynthesis I | 6 | 4 | EC.2.1.2.3 EC.3.5.4.10 EC.4.3.2.2 EC.6.3.2.6 |
| PWY-6124 | inosine-5'-phosphate biosynthesis II | 5 | 4 | EC.2.1.2.3 EC.3.5.4.10 EC.4.3.2.2 EC.6.3.2.6 |
| PWY-6130 | glycerol degradation III | 2 | 1 | EC.4.2.1.30 |
| PWY-6142 | gluconeogenesis II (*Methanobacterium thermoautotrophicum*) | 12 | 7 | EC.1.2.7.1 EC.2.7.1.40 EC.4.1.2.13 EC.4.2.1.1 EC.4.2.1.11 EC.5.3.1.1 EC.5.3.1.9 |
| PWY-6147 | 6-hydroxymethyl-dihydropterin diphosphate biosynthesis I | 5 | 3 | EC.2.7.6.3 EC.3.5.4.16 EC.4.1.2.25 |
| PWY-6164 | 3-dehydroquinate biosynthesis I | 2 | 1 | EC.2.5.1.54 |
| PWY-6168 | flavin biosynthesis III (fungi) | 8 | 2 | EC.3.5.4.25 EC.4.1.99.12 |
| PWY-622 | starch biosynthesis | 9 | 4 | EC.2.4.1.18 EC.2.7.7.27 EC.5.3.1.9 EC.5.4.2.2 |
| PWY-6221 | 2-chlorobenzoate degradation | 1 | 1 | EC.1.14.12.13 |
| PWY-6281 | L-selenocysteine biosynthesis II (archaea and eukaryotes) | 4 | 1 | EC.6.1.1.11 |
| PWY-6282 | palmitoleate biosynthesis I (from (5Z)-dodec-5-enoate) | 5 | 3 | EC.1.1.1.100 EC.1.3.1.9 EC.4.2.1.59 |
| PWY-6303 | methyl indole-3-acetate interconversion | 2 | 1 | EC.3.1.1.1 |
| PWY-6305 | putrescine biosynthesis IV | 4 | 2 | EC.4.1.1.17 EC.4.1.1.19 |
| PWY-6333 | acetaldehyde biosynthesis I | 2 | 1 | EC.1.1.1.1 |
| PWY-6348 | phosphate acquisition | 1 | 1 | EC.3.1.3.2 |
| PWY-6357 | phosphate utilization in cell wall regeneration | 1 | 1 | EC.3.1.3.2 |
| PWY-6375 | desferrioxamine E biosynthesis | 2 | 1 | EC.4.1.1.18 |
| PWY-6376 | desferrioxamine B biosynthesis | 1 | 1 | EC.4.1.1.18 |
| PWY-6381 | bisucaberin biosynthesis | 1 | 1 | EC.4.1.1.18 |
| PWY-6386 | UDP-*N*-acetylmuramoyl-pentapeptide biosynthesis II (lysine-containing) | 8 | 2 | EC.2.5.1.7 EC.6.3.2.8 |
| PWY-6389 | (*S*)-acetoin biosynthesis | 2 | 1 | EC.2.2.1.6 |
| PWY-6435 | 4-hydroxybenzoate biosynthesis V | 5 | 4 | EC.1.1.1.35 EC.2.3.1.16 EC.3.1.2.23 EC.4.2.1.17 |
| PWY-6466 | pyridoxal 5'-phosphate biosynthesis II | 1 | 1 | EC.4.3.3.6 |
| PWY-6471 | peptidoglycan biosynthesis IV (Enterococcus faecium) | 4 | 3 | EC.2.4.1.129 EC.2.7.8.13 EC.3.4.16.4 |
| PWY-6481 | L-dopachrome biosynthesis | 1 | 1 | EC.1.14.18.1 |
| PWY-6483 | ceramide degradation | 1 | 1 | EC.3.5.1.23 |
| PWY-6510 | methanol oxidation to formaldehyde II | 2 | 1 | EC.1.1.1.1 |
| PWY-6523 | nitrite-dependent anaerobic methane oxidation | 2 | 1 | EC.1.7.2.1 |
| PWY-6527 | stachyose degradation | 8 | 3 | EC.2.7.7.9 EC.3.2.1.22 EC.5.1.3.2 |
| PWY-6536 | 4-aminobutanoate degradation III | 2 | 1 | EC.1.2.1.16 |
| PWY-6545 | pyrimidine deoxyribonucleotides *de novo* biosynthesis III | 9 | 4 | EC.1.17.4.1 EC.2.1.1.148 EC.2.7.4.6 EC.3.6.1.15 |
| PWY-6549 | L-glutamine biosynthesis III | 8 | 5 | EC.1.1.1.42 EC.1.4.7.1 EC.2.3.3.1 EC.2.7.9.1 EC.6.3.1.2 |
| PWY-6559 | spermidine biosynthesis II | 4 | 2 | EC.1.2.1.11 EC.2.7.2.4 |
| PWY-6580 | phosphatidylinositol biosynthesis I (bacteria) | 2 | 1 | EC.5.5.1.4 |
| PWY-6583 | pyruvate fermentation to butanol I | 7 | 4 | EC.1.1.1.35 EC.1.2.7.1 EC.1.3.8.1 EC.2.3.1.9 |
| PWY-6587 | pyruvate fermentation to ethanol III | 3 | 2 | EC.1.1.1.1 EC.1.2.7.1 |
| PWY-66 | GDP-L-fucose biosynthesis I (from GDP-D-mannose) | 2 | 2 | EC.1.1.1.271 EC.4.2.1.47 |
| PWY-6609 | adenine and adenosine salvage III | 3 | 1 | EC.3.5.4.4 |
| PWY-6614 | tetrahydrofolate biosynthesis | 3 | 2 | EC.1.5.1.3 EC.2.5.1.15 |
| PWY-6692 | Fe(II) oxidation | 4 | 3 | EC.1.10.2.2 EC.1.6.5.3 EC.1.9.3.1 |
| PWY-6700 | queuosine biosynthesis | 4 | 1 | EC.2.4.2.29 |
| PWY-6728 | methylaspartate cycle | 16 | 12 | EC.1.1.1.37 EC.1.1.1.42 EC.1.3.5.1 EC.1.4.1.2 EC.2.3.3.1 EC.2.3.3.9 EC.4.1.3.24 EC.4.2.1.2 EC.5.1.99.1 EC.5.4.99.2 EC.6.2.1.5 EC.6.4.1.3 |
| PWY-6748 | nitrate reduction VII (denitrification) | 4 | 2 | EC.1.7.2.1 EC.1.7.2.4 |
| PWY-6749 | CMP-legionaminate biosynthesis I | 9 | 1 | EC.2.6.1.16 |
| PWY-6780 | hydrogen production VI | 2 | 1 | EC.1.2.99.2 |
| PWY-6807 | xyloglucan degradation II (exoglucanase) | 4 | 1 | EC.3.2.1.23 |
| PWY-6808 | dTDP-D-forosamine biosynthesis | 4 | 2 | EC.2.7.7.24 EC.4.2.1.46 |
| PWY-6823 | molybdenum cofactor biosynthesis | 7 | 3 | EC.2.8.1.12 EC.2.8.1.7 EC.4.1.99.18 |
| PWY-6837 | fatty acid beta-oxidation V (unsaturated, odd number, di-isomerase-dependent) | 4 | 2 | EC.1.3.1.34 EC.5.3.3.8 |
| PWY-6840 | homoglutathione biosynthesis | 2 | 1 | EC.6.3.2.2 |
| PWY-6854 | ethylene biosynthesis III (microbes) | 3 | 1 | EC.1.15.1.1 |
| PWY-6857 | retinol biosynthesis | 5 | 2 | EC.3.1.1.1 EC.3.1.1.3 |
| PWY-6871 | 3-methylbutanol biosynthesis | 3 | 2 | EC.1.1.1.1 EC.2.3.3.13 |
| PWY-6886 | 1-butanol autotrophic biosynthesis | 5 | 3 | EC.2.7.1.40 EC.2.7.2.3 EC.4.2.1.11 |
| PWY-6901 | superpathway of glucose and xylose degradation | 8 | 5 | EC.1.2.1.12 EC.2.2.1.1 EC.2.7.1.40 EC.2.7.2.3 EC.4.2.1.11 |
| PWY-6902 | chitin degradation II | 2 | 1 | EC.3.2.1.52 |
| PWY-6932 | selenate reduction | 2 | 1 | EC.2.7.7.4 |
| PWY-6936 | seleno-amino acid biosynthesis | 5 | 2 | EC.2.3.1.30 EC.2.5.1.47 |
| PWY-6945 | cholesterol degradation to androstenedione I (cholesterol oxidase) | 12 | 4 | EC.1.1.1.35 EC.2.3.1.16 EC.2.3.1.176 EC.5.3.3.1 |
| PWY-695 | abscisic acid biosynthesis | 5 | 1 | EC.1.13.11.51 |
| PWY-6952 | glycerophosphodiester degradation | 2 | 1 | EC.1.1.5.3 |
| PWY-6963 | ammonia assimilation cycle I | 2 | 2 | EC.1.4.1.14 EC.6.3.1.2 |
| PWY-6964 | ammonia assimilation cycle II | 2 | 2 | EC.1.4.7.1 EC.6.3.1.2 |
| PWY-6969 | TCA cycle V (2-oxoglutarate:ferredoxin oxidoreductase) | 11 | 9 | EC.1.1.1.37 EC.1.1.1.42 EC.1.2.7.3 EC.1.3.5.1 EC.2.3.3.1 EC.2.3.3.9 EC.4.1.3.1 EC.4.2.1.2 EC.6.2.1.5 |
| PWY-6987 | lipoate biosynthesis and incorporation III (Bacillus) | 3 | 1 | EC.2.8.1.8 |
| PWY-7003 | glycerol degradation to butanol | 11 | 7 | EC.1.2.1.12 EC.1.2.7.1 EC.2.3.1.9 EC.2.7.1.40 EC.2.7.2.3 EC.4.2.1.11 EC.5.3.1.1 |
| PWY-7013 | L-1,2-propanediol degradation | 5 | 2 | EC.1.1.1.1 EC.4.2.1.28 |
| PWY-7084 | nitrifier denitrification | 4 | 2 | EC.1.7.2.1 EC.1.7.2.4 |
| PWY-7089 | taxiphyllin bioactivation | 1 | 1 | EC.3.2.1.21 |
| PWY-7092 | neolinustatin bioactivation | 2 | 1 | EC.3.2.1.21 |
| PWY-7094 | fatty acid salvage | 6 | 4 | EC.1.1.1.35 EC.2.3.1.16 EC.4.2.1.17 EC.6.2.1.3 |
| PWY-7096 | triclosan resistance | 1 | 1 | EC.1.3.1.9 |
| PWY-7111 | pyruvate fermentation to isobutanol (engineered) | 5 | 4 | EC.1.1.1.1 EC.1.1.1.86 EC.2.2.1.6 EC.4.2.1.9 |
| PWY-7115 | C4 photosynthetic carbon assimilation cycle, NAD-ME type | 8 | 5 | EC.1.1.1.37 EC.1.1.1.39 EC.2.6.1.1 EC.2.7.9.1 EC.4.2.1.1 |
| PWY-7117 | C4 photosynthetic carbon assimilation cycle, PEPCK type | 8 | 5 | EC.1.1.1.40 EC.2.6.1.1 EC.2.7.9.1 EC.4.1.1.49 EC.4.2.1.1 |
| PWY-7118 | chitin degradation to ethanol | 7 | 4 | EC.1.1.1.1 EC.1.1.1.39 EC.2.3.3.9 EC.6.2.1.1 |
| PWY-7119 | sphingolipid recycling and degradation (yeast) | 5 | 2 | EC.3.5.1.23 EC.4.1.2.27 |
| PWY-7176 | UTP and CTP *de novo* biosynthesis | 4 | 4 | EC.2.7.4.14 EC.2.7.4.22 EC.2.7.4.6 EC.6.3.4.2 |
| PWY-7177 | UTP and CTP dephosphorylation II | 2 | 1 | EC.6.3.4.2 |
| PWY-7179 | purine deoxyribonucleosides degradation I | 2 | 1 | EC.3.5.4.4 |
| PWY-7179-1 | purine deoxyribonucleosides degradation | 2 | 1 | EC.3.5.4.4 |
| PWY-7180 | 2'-deoxy-α-D-ribose 1-phosphate degradation | 3 | 1 | EC.4.1.2.4 |
| PWY-7187 | pyrimidine deoxyribonucleotides *de novo* biosynthesis II | 9 | 2 | EC.2.7.4.6 EC.3.5.4.13 |
| PWY-7205 | CMP phosphorylation | 3 | 2 | EC.2.7.4.14 EC.2.7.4.6 |
| PWY-7214 | baicalein degradation (hydrogen peroxide detoxification) | 2 | 1 | EC.1.11.1.7 |
| PWY-7216 | (R)- and (S)-3-hydroxybutanoate biosynthesis | 3 | 3 | EC.1.1.1.35 EC.1.1.1.36 EC.2.3.1.9 |
| PWY-7218 | photosynthetic 3-hydroxybutanoate biosynthesis (engineered) | 4 | 2 | EC.2.7.1.40 EC.4.2.1.11 |
| PWY-7219 | adenosine ribonucleotides *de novo* biosynthesis | 4 | 3 | EC.2.7.4.3 EC.4.3.2.2 EC.6.3.4.4 |
| PWY-7220 | adenosine deoxyribonucleotides *de novo* biosynthesis II | 3 | 2 | EC.1.17.4.1 EC.2.7.4.6 |
| PWY-7221 | guanosine ribonucleotides *de novo* biosynthesis | 4 | 3 | EC.1.1.1.205 EC.2.7.4.6 EC.6.3.5.2 |
| PWY-7222 | guanosine deoxyribonucleotides *de novo* biosynthesis II | 3 | 2 | EC.1.17.4.1 EC.2.7.4.6 |
| PWY-7226 | guanosine deoxyribonucleotides *de novo* biosynthesis I | 2 | 2 | EC.1.17.4.1 EC.2.7.4.6 |
| PWY-7227 | adenosine deoxyribonucleotides *de novo* biosynthesis | 2 | 2 | EC.1.17.4.1 EC.2.7.4.6 |
| PWY-723 | alkylnitronates degradation | 2 | 1 | EC.1.13.12.16 |
| PWY-7234 | inosine-5'-phosphate biosynthesis III | 6 | 3 | EC.3.5.4.10 EC.4.3.2.2 EC.6.3.2.6 |
| PWY-7250 | [2Fe-2S] iron-sulfur cluster biosynthesis | 2 | 1 | EC.2.8.1.7 |
| PWY-7254 | TCA cycle VII (acetate-producers) | 7 | 4 | EC.1.1.1.42 EC.1.3.5.1 EC.2.3.3.1 EC.4.2.1.2 |
| PWY-7279 | aerobic respiration II (cytochrome c) (yeast) | 4 | 3 | EC.1.10.2.2 EC.1.3.5.1 EC.1.9.3.1 |
| PWY-7291 | oleate β-oxidation (isomerase-dependent, yeast) | 2 | 1 | EC.5.3.3.8 |
| PWY-7299 | progesterone biosynthesis | 2 | 1 | EC.5.3.3.1 |
| PWY-7307 | oleate β-oxidation (reductase-dependent, yeast) | 2 | 1 | EC.1.3.1.34 |
| PWY-7308 | acrylonitrile degradation I | 2 | 1 | EC.3.5.1.4 |
| PWY-7312 | dTDP-D-β-fucofuranose biosynthesis | 4 | 2 | EC.2.7.7.24 EC.4.2.1.46 |
| PWY-7315 | dTDP-*N*-acetylthomosamine biosynthesis | 4 | 2 | EC.2.7.7.24 EC.4.2.1.46 |
| PWY-7316 | dTDP-*N*-acetylviosamine biosynthesis | 4 | 2 | EC.2.7.7.24 EC.4.2.1.46 |
| PWY-7328 | superpathway of UDP-glucose-derived O-antigen building blocks biosynthesis | 2 | 1 | EC.5.1.3.2 |
| PWY-7335 | UDP-*N*-acetyl-α-D-mannosaminouronate biosynthesis | 2 | 1 | EC.5.1.3.14 |
| PWY-7344 | UDP-D-galactose biosynthesis | 1 | 1 | EC.5.1.3.2 |
| PWY-7346 | UDP-α-D-glucuronate biosynthesis (from UDP-glucose) | 1 | 1 | EC.1.1.1.22 |
| PWY-7380 | biotin biosynthesis from 8-amino-7-oxononanoate II | 3 | 1 | EC.2.8.1.6 |
| PWY-7381 | lipoate biosynthesis and incorporation (glycine cleavage system, yeast) | 2 | 1 | EC.2.8.1.8 |
| PWY-7383 | anaerobic energy metabolism (invertebrates, cytosol) | 6 | 4 | EC.1.1.1.37 EC.2.6.1.1 EC.2.7.1.40 EC.4.1.1.32 |
| PWY-7384 | anaerobic energy metabolism (invertebrates, mitochondrial) | 11 | 6 | EC.1.1.1.39 EC.4.2.1.2 EC.5.1.99.1 EC.5.4.99.2 EC.6.2.1.5 EC.6.4.1.3 |
| PWY-7385 | 1,3-propanediol biosynthesis (engineered) | 8 | 4 | EC.2.7.1.11 EC.4.1.2.13 EC.4.2.1.30 EC.5.3.1.9 |
| PWY-7391 | isoprene biosynthesis II (engineered) | 8 | 3 | EC.2.3.1.9 EC.4.1.1.33 EC.5.3.3.2 |
| PWY-7396 | butanol and isobutanol biosynthesis (engineered) | 7 | 2 | EC.1.1.1.1 EC.1.1.1.85 |
| PWY-7400 | L-arginine biosynthesis IV (archaebacteria) | 9 | 6 | EC.1.2.1.38 EC.2.1.3.3 EC.2.7.2.8 EC.4.3.2.1 EC.6.3.4.5 EC.6.3.5.5 |
| PWY-7413 | dTDP-6-deoxy-α-D-allose biosynthesis | 4 | 2 | EC.2.7.7.24 EC.4.2.1.46 |
| PWY-7417 | phospholipid remodeling (phosphatidate, yeast) | 2 | 1 | EC.2.3.1.51 |
| PWY-7459 | kojibiose degradation | 2 | 1 | EC.2.4.1.230 |
| PWY-7494 | choline degradation IV | 1 | 1 | EC.1.2.1.8 |
| PWY-7518 | atromentin biosynthesis | 2 | 1 | EC.2.6.1.57 |
| PWY-7539 | 6-hydroxymethyl-dihydropterin diphosphate biosynthesis III (Chlamydia) | 5 | 3 | EC.2.7.6.3 EC.3.5.4.25 EC.4.1.2.25 |
| PWY-7557 | dehydrodiconiferyl alcohol degradation | 4 | 2 | EC.1.1.1.1 EC.1.2.1.3 |
| PWY-7573 | GDP-mycosamine biosynthesis | 2 | 1 | EC.4.2.1.47 |
| PWY-7586 | β-1,4-D-mannosyl-*N*-acetyl-D-glucosamine degradation | 3 | 2 | EC.5.3.1.8 EC.5.4.2.8 |
| PWY-7589 | palmitoleate biosynthesis III (cyanobacteria) | 2 | 1 | EC.2.3.1.51 |
| PWY-7661 | protein *N*-glycosylation (*Haloferax volcanii*) | 4 | 1 | EC.2.4.1.83 |
| PWY-7663 | gondoate biosynthesis (anaerobic) | 4 | 3 | EC.1.1.1.100 EC.1.3.1.9 EC.4.2.1.59 |
| PWY-7664 | oleate biosynthesis IV (anaerobic) | 7 | 5 | EC.1.1.1.100 EC.1.3.1.9 EC.2.3.1.85 EC.4.2.1.59 EC.5.3.3.14 |
| PWY-801 | L-homocysteine and L-cysteine interconversion | 1 | 1 | EC.4.2.1.22 |
| PWY-881 | trehalose biosynthesis II | 2 | 1 | EC.3.1.3.12 |
| PWY0-1021 | L-alanine biosynthesis III | 1 | 1 | EC.2.8.1.7 |
| PWY0-1221 | putrescine degradation II | 3 | 1 | EC.6.3.1.11 |
| PWY0-1261 | anhydromuropeptides recycling | 10 | 3 | EC.2.3.1.157 EC.3.2.1.52 EC.3.5.1.28 |
| PWY0-1264 | biotin-carboxyl carrier protein assembly | 3 | 2 | EC.6.3.4.14 EC.6.4.1.2 |
| PWY0-1275 | lipoate biosynthesis and incorporation II | 2 | 1 | EC.2.8.1.8 |
| PWY0-1299 | arginine dependent acid resistance | 1 | 1 | EC.4.1.1.19 |
| PWY0-1301 | melibiose degradation | 1 | 1 | EC.3.2.1.22 |
| PWY0-1303 | aminopropylcadaverine biosynthesis | 2 | 1 | EC.4.1.1.18 |
| PWY0-1305 | glutamate dependent acid resistance | 1 | 1 | EC.4.1.1.15 |
| PWY0-1312 | acetate formation from acetyl-CoA I | 2 | 1 | EC.2.3.1.8 |
| PWY0-1313 | acetate conversion to acetyl-CoA | 1 | 1 | EC.6.2.1.1 |
| PWY0-1319 | CDP-diacylglycerol biosynthesis II | 4 | 2 | EC.1.1.1.94 EC.2.3.1.51 |
| PWY0-1329 | succinate to cytochrome *bo* oxidase electron transfer | 2 | 1 | EC.1.3.5.1 |
| PWY0-1334 | NADH to cytochrome *bd* oxidase electron transfer I | 2 | 1 | EC.1.6.5.3 |
| PWY0-1335 | NADH to cytochrome *bo* oxidase electron transfer I | 2 | 1 | EC.1.6.5.3 |
| PWY0-1353 | succinate to cytochrome *bd* oxidase electron transfer | 2 | 1 | EC.1.3.5.1 |
| PWY0-1415 | superpathway of heme biosynthesis from uroporphyrinogen-III | 4 | 3 | EC.1.3.3.3 EC.4.1.1.37 EC.4.99.1.1 |
| PWY0-1479 | tRNA processing | 5 | 1 | EC.2.7.7.56 |
| PWY0-1561 | glycerol-3-phosphate to cytochrome *bo* oxidase electron transfer | 2 | 1 | EC.1.1.5.3 |
| PWY0-1581 | nitrate reduction IX (dissimilatory) | 2 | 1 | EC.1.1.5.3 |
| PWY0-1582 | glycerol-3-phosphate to fumarate electron transfer | 2 | 1 | EC.1.1.5.3 |
| PWY0-1584 | nitrate reduction X (periplasmic, dissimilatory) | 2 | 2 | EC.1.1.5.3 EC.1.7.99.4 |
| PWY0-1586 | peptidoglycan maturation (*meso*-diaminopimelate containing) | 3 | 2 | EC.2.4.1.129 EC.3.4.16.4 |
| PWY0-321 | phenylacetate degradation I (aerobic) | 9 | 3 | EC.1.1.1.35 EC.1.14.13.149 EC.4.2.1.17 |
| PWY0-461 | L-lysine degradation I | 1 | 1 | EC.4.1.1.18 |
| PWY0-501 | lipoate biosynthesis and incorporation I | 2 | 1 | EC.2.8.1.8 |
| PWY0-662 | PRPP biosynthesis I | 1 | 1 | EC.2.7.6.1 |
| PWY0-823 | L-arginine degradation III (arginine decarboxylase/agmatinase pathway) | 2 | 1 | EC.4.1.1.19 |
| PWY0-862 | (5Z)-dodec-5-enoate biosynthesis | 7 | 5 | EC.1.1.1.100 EC.1.3.1.9 EC.2.3.1.85 EC.4.2.1.59 EC.5.3.3.14 |
| PWY1-3 | polyhydroxybutanoate biosynthesis | 3 | 2 | EC.1.1.1.36 EC.2.3.1.9 |
| PWY1F-353 | glycine betaine biosynthesis III (plants) | 2 | 1 | EC.1.2.1.8 |
| PWY3O-210 | L-glutamate degradation IX (via 4-aminobutanoate) | 1 | 1 | EC.4.1.1.15 |
| PWY490-4 | L-asparagine biosynthesis III (tRNA-dependent) | 3 | 1 | EC.6.3.5.6 |
| PWY66-161 | oxidative ethanol degradation III | 2 | 2 | EC.1.2.1.3 EC.6.2.1.1 |
| PWY66-162 | ethanol degradation IV | 3 | 3 | EC.1.11.1.6 EC.1.2.1.3 EC.6.2.1.1 |
| PWY66-201 | nicotine degradation IV | 9 | 1 | EC.1.14.13.8 |
| PWY66-21 | ethanol degradation II | 3 | 3 | EC.1.1.1.1 EC.1.2.1.3 EC.6.2.1.1 |
| PWY66-368 | ketolysis | 3 | 2 | EC.2.3.1.9 EC.2.8.3.5 |
| PWY66-389 | phytol degradation | 4 | 3 | EC.1.1.1.1 EC.1.2.1.3 EC.6.2.1.3 |
| PWY66-391 | fatty acid β-oxidation VI (peroxisome) | 7 | 4 | EC.1.1.1.35 EC.2.3.1.16 EC.4.2.1.17 EC.6.2.1.3 |
| PWY66-398 | TCA cycle III (animals) | 10 | 7 | EC.1.1.1.37 EC.1.2.4.2 EC.1.3.5.1 EC.2.3.1.61 EC.2.3.3.1 EC.4.2.1.2 EC.6.2.1.5 |
| PWY66-399 | gluconeogenesis III | 13 | 9 | EC.1.1.1.37 EC.1.2.1.12 EC.2.7.2.3 EC.3.1.3.11 EC.4.1.1.32 EC.4.1.2.13 EC.4.2.1.11 EC.5.3.1.1 EC.5.3.1.9 |
| PWY66-400 | glycolysis VI (metazoan) | 11 | 8 | EC.1.2.1.12 EC.2.7.1.11 EC.2.7.1.40 EC.2.7.2.3 EC.4.1.2.13 EC.4.2.1.11 EC.5.3.1.1 EC.5.3.1.9 |
| PWY66-428 | L-threonine degradation V | 1 | 1 | EC.4.3.1.19 |
| PWYG-321 | mycolate biosynthesis | 16 | 7 | EC.1.1.1.100 EC.1.3.1.9 EC.2.3.1.179 EC.3.1.3.12 EC.4.2.1.59 EC.5.3.3.14 EC.6.4.1.3 |
| PWYQT-4429 | CO2 fixation into oxaloacetate (anaplerotic) | 2 | 1 | EC.4.2.1.1 |
| PYRIDOXSYN-PWY | pyridoxal 5'-phosphate biosynthesis I | 6 | 3 | EC.1.4.3.5 EC.2.2.1.7 EC.2.6.1.52 |
| PYRUVDEHYD-PWY | pyruvate decarboxylation to acetyl CoA | 3 | 3 | EC.1.2.4.1 EC.1.8.1.4 EC.2.3.1.12 |
